# Predictive values of carotid high-resolution magnetic resonance imaging for large embolus shedding in carotid artery stenting

**DOI:** 10.3906/sag-1811-40

**Published:** 2019-05-29

**Authors:** Xingyue ZHENG, Erling WANG, Xiaoqian GUO, Chan GU, Zhongrui YAN, Peng YAN, Shan LI, Chuanqiang QU, Xiyun RUAN, Jifeng LI

**Affiliations:** 1 Department of Neurology, Shandong Provincial Hospital Affiliated to Shandong First Medical University, Jinan, China; 2 Department of Neurology, Jining No.1 People’s Hospital, Jining, China

**Keywords:** Carotid stenosis, stent, high-resolution magnetic resonance imaging, plaque

## Abstract

**Background/aim:**

Embolus shedding is one of the important complications in carotid artery stenting (CAS). Carotid high-resolution magnetic resonance imaging (HR-MRI) is often used to directly reflect important biological characteristics, such as plaque size and composition, as well as the structure of the carotid artery wall. The aim of this study was to investigate the predictive values of carotid HR-MRI for large embolus shedding in CAS.

**Materials and methods:**

In total, 195 patients with carotid stenosis were enrolled. Preoperative carotid HR-MRI was performed to define the nature of the carotid plaques. CAS was performed in all patients, and intraoperative embolic protection devices were used to collect the shed emboli. According to the diameter and number of shed emboli, the patients were divided into the small-embolus group (group X) and large-embolus group (group Y). Logistic regression analysis was used to analyze the risk factors of large embolus shedding.

**Results:**

Group Y included 58 patients, and group X included 137 patients. Age, stenosis length, smoking, and ≥3 transient cerebral ischemic attacks were risk factors for large embolus shedding. Two cases of shed large emboli developed from stable plaques, and 56 cases of large emboli developed from vulnerable plaques. When vulnerable plaques were associated with more risk factors, the incidences of large embolus shedding in cases with vulnerable plaques combined with 0, 1, 2, 3, and 4 risk factors were 44 % (4/9), 68.1% (15/22), 72.2% (13/18), 76.5% (13/17), and 84.6% (11/13), respectively.

**Conclusion:**

Carotid HR-MRI can predict the incidence of large embolus shedding in CAS.

## 1. Introduction

Cerebral infarction (CI) is one of the main causes for the death and disability in elderly individuals, and carotid stenosis is an important risk factor for CI. It has been reported that about 26%–28% of patients with a carotid stenosis degree of 70%–90% as well as cerebral ischemic symptoms will develop CI within 1 year [1], and carotid stenosis-induced CI accounts for 20% of CI cases [2]. Carotid artery stenting (CAS) and carotid endarterectomy (CEA) are the two main surgical methods used to manage carotid stenosis [3], and CEA has always been considered as the “gold standard” treatment for carotid artery stenosis. However, CAS has progressively become the new technique that is most likely to replace CEA [4,5]. One serious complication in CAS is lesion plaque shedding, resulting in CI [6]. There still exist wide controversies regarding whether applying cerebral protection devices can reduce the occurrence of intra-CAS CI. Some reports indicate that the use of embolic protection devices (EPDs) can effectively reduce the occurrence of embolism complications [6,7], but other reports indicate that the use of EPDs not only effectively reduce, but also increase surgery costs and protection device-induced complications, such as vascular spasm and vascular dissection [8–10]. The most important complication that causes death and disability in CAS is intraoperative embolus shedding-induced vascular embolization [6].

Carotid high-resolution magnetic resonance imaging (HR-MRI) can directly reflect important biological characteristics such as plaque size and composition, as well as the structure of the carotid artery wall. Carotid HR-MRI has unique advantages in evaluating different types of atherosclerotic plaques, especially in evaluating the stability of vulnerable plaques, compared with those of other methods [11]. Whether carotid HR-MRI can be used to assess patients’ carotid plaques to predict the incidence of embolus shedding in advance — thus providing a reliable clinical basis for the necessity of applying EPDs in high-risk patients — remains a question. This study intended to find the risk factors for the occurrence of large emboli combined with carotid HR-MRI to provide clinical judgment of carotid plaque types before CAS, predict the incidence of intraoperative embolus shedding, and provide a basis for the application of EPDs.

## 2. Patients and methods

### 2.1. Patients

A total of 195 patients who underwent CAS in the Affiliated Provincial Hospital between October 2012 and December 2015 were enrolled. The inclusion criteria were as follows: carotid stenosis ≥70%, symptomatic or asymptomatic poor collateral circulation, and age >45 years. The exclusion criteria were as follows: thrombocytopenia, <100×10^9^/L; leucopenia, <4×10^9^/L; neutropenia; cerebral hemorrhage within nearly 3 months; allergic to aspirin or clopidogrel; severe heart or lung disease; and bead-string-like CAS. If the patient newly developed CI, the surgery was routinely delayed for 3 weeks. If the patient did not present with symptom aggravation within 3 weeks and diffusion-weighted imaging (DWI) or computed tomography (CT) showed good absorption of the new lesion or the lesion was significantly reduced than before, the patients could undergo CAS. The study was supervised by the Medical Ethics Committee of Shandong Provincial Hospital Affiliated to Shandong First Medical University, and all the patients or their legal guardians signed the informed consent.

### 2.2. Perioperative preparation

All the patients underwent digital subtraction angiography using one angiography instrument (Artis zee floor, Siemens, Germany). After local anesthesia was administered, the femoral artery was punctured using the Seldinger method, and one 5F vascular sheath was implanted. The carotid and vertebral arteries underwent arteriography in turn using the catheter. Carotid stenosis was measured according to the criteria of the North American Symptomatic Carotid Endarterectomy Trial [12]. Before CAS, the patients with carotid stenosis >70% selectively underwent carotid HR-MRI (PHILIPS 3.0T, Philips, The Netherlands) with a carotid dedicated 4-channel phased-array surface coil. The system was positioned parallel to the line that connected the anteroposterior edge of foramen magnum. Initial scans were conducted using standard carotid 2D high-resolution MRI sequences in order to obtain the initial position: axial 3D TOF and 2D-BB-MRI placed at the center of the bilateral carotid artery bifurcation and the 2 cm region around the center was subsequently scanned. The plaque composition (lipid-rich core, intraplaque hemorrhage, and calcification) and the surface condition of the fibrous cap of the plaque (complete or broken) in the common carotid artery, carotid bifurcation, and internal carotid artery as references, were observed and used to assess the stability of the plaque, which then were divided into two groups: stable plaques and vulnerable plaques. Stable plaques referred to types VII and VIII and vulnerable plaques referred to types IV, V, and VI in the American Heart Association (AHA) classification [13]. In HR-MRI, vulnerable plaques are defined as the plaque lesions that may progress rapidly to pathogenic lesions or plaques with severe thrombotic tendency, i.e. type IV (atheroma), type V (fibrous poroma), and type VI (complex plaque, intracerebral hemorrhage) in the classification of atherosclerosis (AS) [14–17]. The MRI signal performances of types IV and V of the AHA classification (compared with the sternocleidomastoid muscle at the same level) are as follows: time-of-flight (TOF) images show iso-signal, T1-weighted (T1W) images show iso-signal, T2-weighted (T2W) images show low signal, and proton density weighted images (PDWI) show low signal. The MRI signal performances of type VI [18,19] are as follows: fresh intraplaque hemorrhage and recent hemorrhage. The MRI performances of fresh intraplaque hemorrhage are as follows: TOF images show iso-signal, T1W images show high signal, T2W images show low or iso-signal, and PDWI show low or iso-signal. The MRI performances of recent plague hemorrhage are as follows: TOF images show high signal, T1W images show high signal, T2W images show high signal, and PDWI show high signal.

Stable plaques are type VII (calcified plaque) and type VIII (fibrous plaque) in the classification of AS plaques. The MRI signals of type VII plaques showed low signals in T1WI, T2WI, and PDWI; type VIII plaques showed iso-signal or high signal on T1WI, slightly high or iso-signal on PDWI and slightly high signal or iso-signal on T2WI [20]. As a noninvasive method, HR-MRI can comprehensively evaluate the functions and morphologies of different types of plaques and then determine the potential rupture risks of different types of plaques. Before surgery, 100 mg of aspirin and 75 mg of clopidogrel were administered for at least 3 days.

### 2.3. Procedures of CAS

When performing CAS, the right femoral artery was first punctured using the Seldinger method, and 4000–5000 units of heparin saline (70 units/kg) was then injected together with an intraoperative intravenous push of 2000 units of heparin saline every other hour to ensure the heparinized state of the whole body (even if the clotting time was maintained between 250 and 300 s). One 8F catheter was then inserted into the stenotic vessel with its tip placed 20 mm below the stenosis. In order to ensure that the maximum diameter of the cerebral protection device (Accunet, Abbott, USA and Emboshield NAV6, Abbott, USA) could safely pass through the stenosis, if the residual luminal segment at the stenosis was <2 mm, a 2-mm balloon system (Viatrac, Abbott, USA) was delivered via a micro-wire to preexpand the stenosis.

After EPDs were successfully placed, the appropriate balloons (Acculink, Abbott, USA) were used to preexpand the stenosis, and after the expansion, the appropriate stents (Acculink, Abbott, USA) were selected for CAS. After the stent was released, if the residual vascular lumen was ≥30%, the balloon system was reused for the post-stenting expansion to achieve the purpose of reducing the residual vascular lumen to <30%.

During stent release, the patient’s heart rate may decrease rapidly, so the patient was asked to cough immediately after the stent was released; if the heart rate still increased slowly 10 s later, atropine (maximum dose, 0.5 mg) was intravenously injected immediately. If the intraoperative blood pressure dropped, there was no need to increase the blood pressure immediately, and observation alone could be performed if no symptoms appeared. If the blood pressure dropped to 90/60 mmHg or less, dopamine (3–10 μg·kg^−1^·min^−1^) could be administered to increase the blood pressure and to ensure that the systolic blood pressure was not less than 90 mmHg. Meanwhile, the systolic blood pressure could not exceed 120 mmHg to prevent hypertension-induced high perfusion syndrome. After releasing the stent, EPDs were carefully recycled, marked, and recorded correspondingly for the statistical analysis later.

### 2.4. Postoperative treatment

After CAS, the patient was sent back to the ward for at least 24-h electrocardiogram monitoring; meanwhile, the heart rate and blood pressure were strictly monitored, and pressor or anti-hypertensive agent doses were adjusted according to the blood pressure and heart rate. If intraoperative headache or ineffective limb activity developed, the surgery was discontinued immediately and neurological examination was performed. If positive signs were found, intraoperative C-arm-assisted brain scans were performed. If the above symptoms appeared postoperatively, CT was performed to exclude cerebral hemorrhage.

### 2.5. Out-of-hospital medication

Each patient was orally administered 75 mg of clopidogrel and 100 mg of aspirin routinely for 3 months after discharge. The patients were instructed to return for clinical follow-up in the 1st, 3rd, and 6th months for guidance and adjustment of the medication doses.

### 2.6. Grouping of emboli and patients

**Figure 1:**
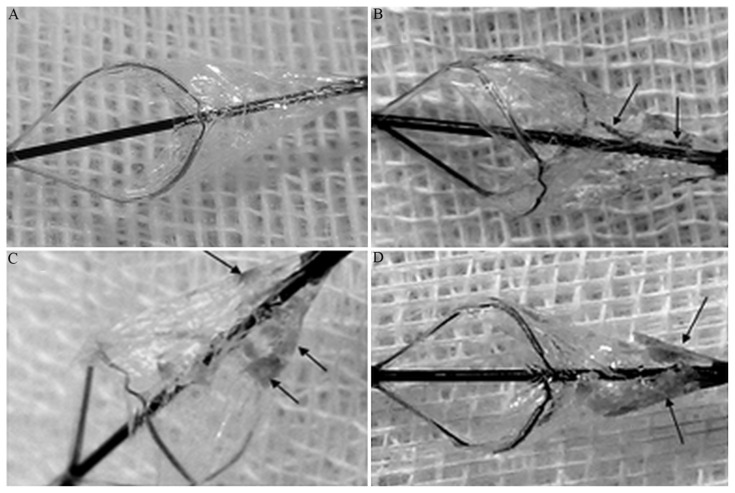
Emboli collected from intraoperative embolus protection devices. The arrows referred to the collected emboli. A: type A, no embolus was found; B: type B, 2 or less emboli with the diameter < 2 mm; C: type C, 3 or more emboli with the diameter < 2 mm; D: type D: more than 1 embolus with the diameter ≥ 2 mm.

The emboli were divided into four types: A, no shed embolus was found within the protection device; B, the embolic diameter within the protection device was <2 mm, and the number was ≤2; C, the embolic diameter within the protection device was <2 mm, but the number was ≥3; and D, the embolic diameter within the protection device was ≥2 mm, and the number was ≥1 (Figure 1). According to the reported criteria [21], types C and D emboli were defined as large emboli and types A and B emboli as small emboli. According to the types of the emboli, the patients were divided into two groups: group X (with types A and B emboli) and group Y (with types C and D emboli). The numbers of large and small emboli were counted when vulnerable plaques were combined with different risk factors to calculate the probability of the occurrence of large emboli.

### 2.7. Statistical analysis

SPSS 17.0 software (SPSSInc., Chicago, IL, USA) was used for the statistical analysis. Logistic regression analysis was used to compare the characteristics and disease history of the patients to determine the risk factors that might result in the occurrence of large emboli during CAS. The p value <0.05 was defined as statistically significant.

## 3. Results

### 3.1. Preoperative carotid HR-MRI manifestations

**Figure 2:**
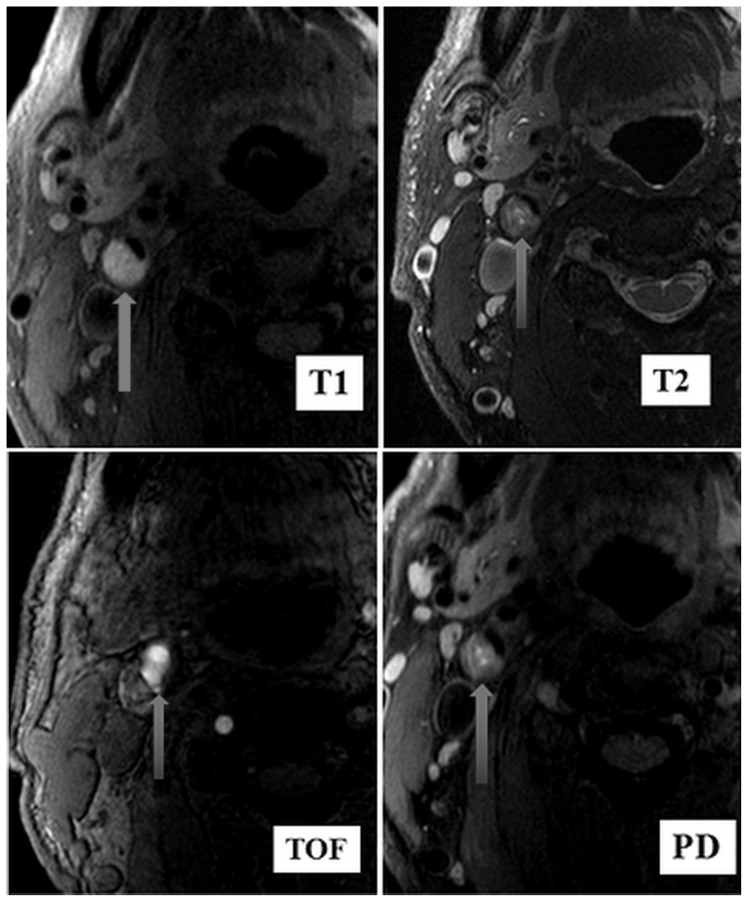
C-HR-MRI revealed vulnerable plaques (type VI: intraplaque hemorrhage). The plaque was presented by the arrow. The C-HR-MRI manifestations of type VI AS (intraplaque hemorrhage) were high-signal T1 and TOF images, as well as high-, iso-, or low-signal T2 and PD images.

Before surgery, carotid HR-MRI showed that, of 195 patients, 116 patients had stable plaques and 79 patients had vulnerable plaques (Figure 2).

### 3.2. Overall treatment outcomes

**Figure 3:**
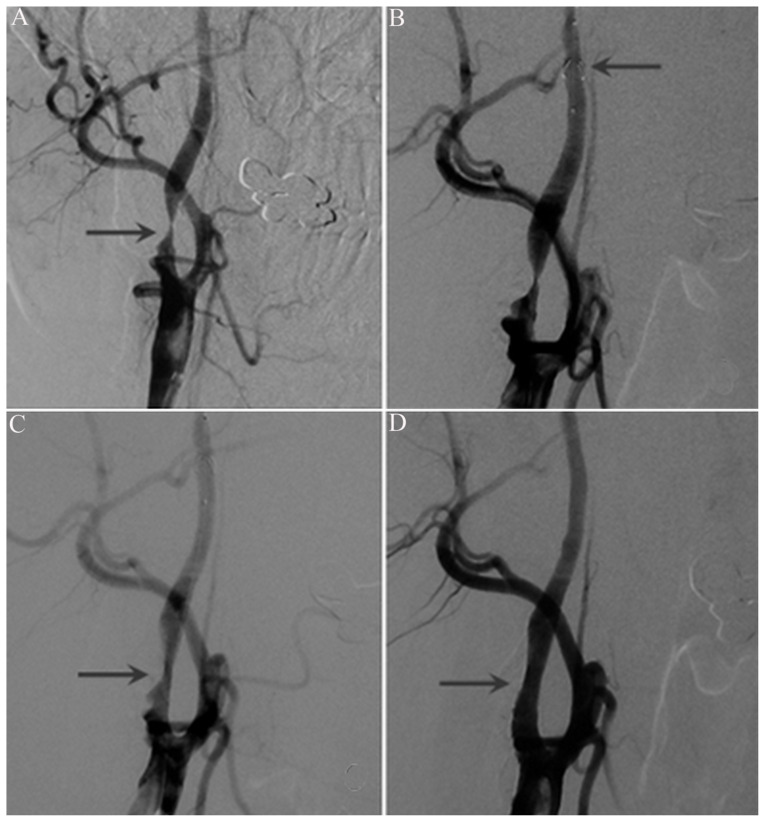
Procedures of CAS. A: The arrow showed severe stenosis at the right internal carotid artery. B: The arrow showed the embolic protection device. C: The arrow showed that stenosis was alleviated after applying balloon dilatation at the severely stenotic site of right carotid artery. D: The arrow showed that the intra-carotid artery stenosis was significantly alleviated after releasing the intra-carotid stent.

A total of 195 patients successfully underwent CAS. The procedure of CAS is shown in Figure 3. During surgery, EPDs were used in all patients. It was difficult to directly withdraw the EPDs in 26 patients. After rotating the neck, swallowing, or pressing the neck, the EPDs were finally successfully withdrawn.

### 3.3. Collection of emboli

After surgery, 58 cases of large emboli and 137 cases of small emboli were collected using EPDs. Among 58 cases of large emboli, 56 cases involved vulnerable plaques and 2 cases involved stable plaques.

### 3.4. Complications

On the day of the surgery, hyperperfusion symptoms occurred in 6 patients. Two patients had intracranial hemorrhage on the day and on the second day of surgery, respectively.

### 3.5. Logistic regression analysis of major embolic risk factors

**Figure 4:**
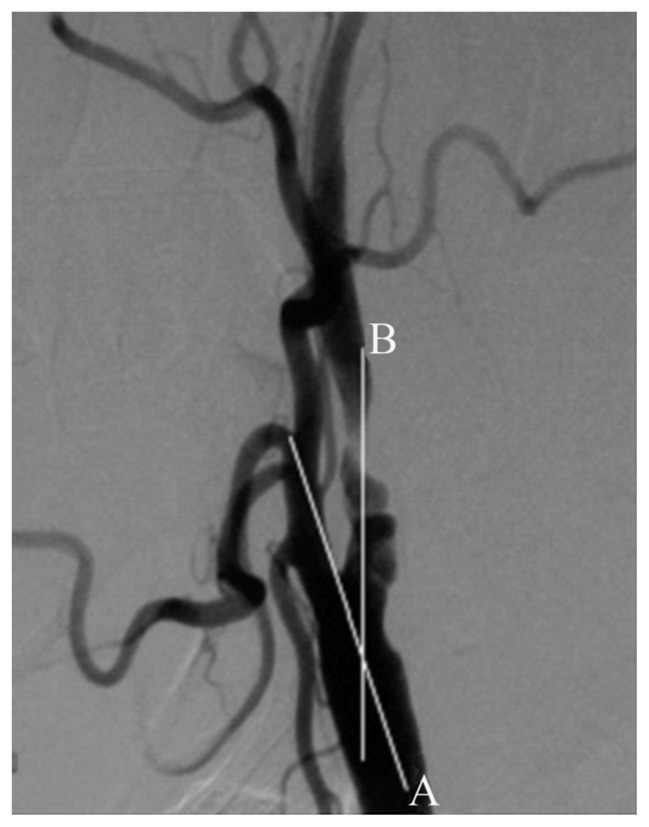
Lateral neck angle: the angle between Line A and B.

Logistic regression analysis of the risk factors of large emboli is shown in Table 1. The lateral neck angle is shown in Figure 4. Logistic regression analysis showed that the age (p = 0.0465), stenosis length (p = 0.0475), smoking (p = 0.0102), and ≥3 preoperative transient cerebral ischemic attacks (p = 0.0172) were the risk factors of large embolus shedding.

### 3.6. Incidences of large embolus shedding for vulnerable plaques combined with risk factors

The incidences of large embolus shedding for vulnerable plaques combined with risk factors are shown in Table 2. The incidences of large embolus shedding in the cases with vulnerable plaques combined with 0, 1, 2, 3, and 4 risk factors were 44% (4/9), 68.1% (15/22), 72.2% (13/18), 76.5% (13/17), and 84.6% (11/13), respectively.

## 4. Discussion

Presently, there exist controversies about the two main surgical methods of carotid stenosis, namely CAS and CEA. However, the Carotid Revascularization Endarterectomy versus Stenting Trial (CREST) shows that complications such as stroke, myocardial infarction, or mortality occurred in the two methods are not significantly different, but CAS has greater advantages for intra-carotid stenosis in high-cervical and intracranial segments [4].

Esposito-Bauer et al. [22] performed carotid HR-MRI in 77 asymptomatic patients with carotid stenosis, and all these patients were followed up for 41.1 months. Nine patients (11.7%) exhibited stenosis-ipsilateral CI, but the patients with stable plaques did not develop cerebrovascular events during the follow-up. Esposito-Bauer et al. [22] showed that carotid HR-MRI has high sensitivity in judging vulnerable plaques and can be used to determine whether patients will develop transient ischemic attack or stroke in the future. The incidence of acute ischemic cerebrovascular events is closely related to vulnerable plaques. Takaya et al. [23] pointed out that the occurrence of ischemic cerebrovascular events in patients with vulnerable plaques is likely to be related to the vulnerable plaque rupture or vulnerable plaque combined with thrombosis. Gröschel et al. [24] retrospectively analyzed 176 patients who underwent CAS, and the results showed that old age (>80 years) and plaque length are risk factors for new infarction. Sayeed et al. [25] studied and analyzed 421 patients who underwent CAS and found that when the plaque length of carotid stenosis is >15 mm, the likelihood of concurrent infarction is greater. This study confirmed that in addition to the age (p = 0.0465), stenosis length (p = 0.0475), and smoking (p = 0.0102), ≥3 preoperative transient ischemic attack episodes (p = 0.0172) are a risk factor for large embolus shedding.

In this study, 26 patients had difficulties recovering with the use of an EPD, which was finally solved after allowing these patients to turn their necks, perform a swallowing action, or pressing the neck. Müller-Hülsbeck et al. [26] indicated that the carotid arteries in which EPDs were applied in pig carotid stenosis models exhibited varying degrees of micro-injuries in the vascular wall. In one clinical analysis by Lian et al. [3], 30 (15.4%) of 195 patients who underwent CAS exhibited difficulties in recovering with the use of EPDs, among whom 29 patients finally recovered after long-term attempts and one patient recovered following EPD removal via surgery. Cremonesi et al. [27] and Shilling et al. [28] also reported cases with difficult EPD removal for devices entering the edge of the stent or those that were stuck in the stent and were finally removed surgically. Lian et al. [3] showed that type VII plaques (namely calcified plagues) or carotid artery torsion angle >80° are risk factors for difficulties in intraoperative EPD recovery. If the above factors exist, it is likely for approximately 15.4% of cases of intra-CAS EPDs to experience retrieval difficulties (13.3% in this study), and surgical removal of EPDs is necessary in rare cases. Based on fluently implementing CAS, assessing the application indications of EPDs and independent risk factors for difficult EPD recovery can exhibit important roles in reducing EPD-induced complications.

Our statistical results showed that when vulnerable plaques were associated with different numbers of risk factors, the probabilities of the incidence of large emboli are different. Given the results of this single-center trial, vulnerable plaques (associated with or without risk factors of large embolus shedding) are important indications for the application of EPDs. Therefore, we recommend that the patients with vulnerable plaques should receive EPDs during CAS. Carotid HR-MRI also showed that 116 of the 195 patients had stable plaques, and finally, 2 large emboli were observed in cases with stable plaques, with an incidence of large emboli of 1.7%. The incidence of large emboli in cases with stable plaques is low, so not applying EPDs can be considered. However, small emboli have clinical uncertainty in patients with stable plaques, so whether patient with stable plaques need EPDs still needs further experimental studies.

There still exist several shortcomings in this study. First, no cranial DWI scanning was performed after CAS, so no preoperative and postoperative DWI comparison can be performed, and no timely diagnosis can be made regarding the existence of asymptomatic infarction. Second, statistical analysis was not performed for the micro-plaques in EPDs, thus resulting in statistical errors. Finally, the MRI scanning time was too long and the number of scanning sequences were too many to be easily accepted by elderly patients.

In conclusion, carotid HR-MRI provides a new imaging method for clinically studying the rupture mechanisms of vulnerable plaques and detecting vulnerable plaques early, which has confirmed its high detection rate and strong specificity toward both vulnerable and stable plaques. Therefore, in combination with patients’ own risk factors, it can be used to predict the incidence of intraoperative embolus shedding.

## Figures and Tables

**Table 1:** Logistic regression analysis of major embolic risk factors.

Index	Small-embolus group (n = 137)	Large-embolus group (n = 58)	P
Age	60.52 ± 5.57	72.49 ± 5.24	0.0465
Sex			0.2199
Male [n(%)]	97 (70.8%)	53 (91.4%)	
Female [n(%)]	38 (29.2%)	5 (8.6%)	
Hypertension [n(%)]	64 (46.7%)	24 (41.4%)	0.3010
Diabetes [n(%)]	45 (32.8%)	27 (46.6%)	0.4654
Stenosis length (mm)	13.44 ± 3.95	19.32 ± 6.18	0.0475
Vascular stenosis rate (%)	79.68 ± 9.38	82.80 ± 9.32	0.4221
Lateral neck angle (Figure 4)	135.27 ± 17.84	149.04 ± 16.05	0.5132
Drinking [n(%)]	75 (54.7%)	38 (65.5%)	0.4534
Smoking [n(%)]	37 (27%)	35 (60.3%)	0.0102
TIA ≥ 3 [n(%)]	25 (18.2%)	40 (69%)	0.0172

TIA, transient cerebral ischemic attack.

**Table 2:** Incidences of large embolus shedding when vulnerable plaques combined with different risk factors.

Number of risk factor	Cases	Large embolus	Small embolus	Incidence of large embolus
0	9	4	5	44.4%
1	22	15	7	68.1%
2	18	13	5	72.2%
3	17	13	4	76.5%
4	13	11	2	84.6%
Total	79	56	23	
